# Sporting Mass Gatherings: Public Health Preparedness and Responsibility of Health Authorities

**Published:** 2018-08-18

**Authors:** Manoochehr Karami

**Affiliations:** ^1^ Research Center for Health Sciences, Hamadan University of Medical Sciences, Hamadan, Iran; ^2^ Department of Epidemiology, School of Public Health, Hamadan University of Medical Sciences, Hamadan, Iran

Mass gathering (MG) is defined as "any type of organized or spontaneous occasion that attracts sufficient numbers of peoples to strain the planning and response sources of the city or nation hosting the event" ^1^. Published literature addressed public health aspects and associated risks with MG and necessity of public health preparedness as the responsibility of public health authorities ^2-5^. There are several concerns regarding MG from the viewpoint of public health authorities including risk of spreading infectious diseases by participants worldwide ^6^.

Different types of MG include religious, cultural and sporting ones are held by hosting countries throughout word. Recently the 21^st^ FIFA world cup, 2018, was hosted by Moscow, Russian Federation from Jun 14, 2018, to Jul 15, 2018. This large sporting event attracted large number of visitors from around the world. Public health authorities of hosting countries are responsible for public health events including potential outbreaks, which may occur during such type of MG. As WHO recommended, hosts of international and large MG should improve public health preparedness to prevent and mitigate the impact of potential public health emergencies with international concerns such as Middle East respiratory syndrome coronavirus (MERS-CoV) and similar infectious diseases ^4,7^.

In the process of a MG, the concentration of people may impact the local health systems. Public health authorities of such MG host country should follow critical proposed elements, including implementation syndromic surveillance systems or enhances surveillance systems, educating participants on occurred health events and other activities according to relevant guidelines in light of situation^4,7-9^. Moreover, the spread of some health events which originated from hosted country may extend to home community once participants return to their own countries. This consideration is a serious challenge to countries, which departed MG participants when they affected by an infectious disease with long incubation period ^6^.

Public health authorities of hosting countries of both sporting and religious MG are advised to consider the risk of spreading infectious diseases especially MERS-CoV. As a specific suggestion regarding Hamadan 2018 event, Hamedan capital of Asian tourism, public health authorities of Hamadan Province are suggested to consider similar requirements. In case of sporting events, it is strongly recommended to use FIFA or other federation's website as an opportunity to educate interested visitors on hand hygiene, respiratory hygiene practices and infection prevention measures.
